# Lightweight Machine-Learning Model for Efficient Design of Graphene-Based Microwave Metasurfaces for Versatile Absorption Performance

**DOI:** 10.3390/nano13020329

**Published:** 2023-01-12

**Authors:** Nengfu Chen, Chong He, Weiren Zhu

**Affiliations:** Department of Electronic Engineering, Shanghai Jiao Tong University, Shanghai 200240, China

**Keywords:** graphene, metasurface design, machine learning, microwave absorption

## Abstract

Graphene, as a widely used nanomaterial, has shown great flexibility in designing optically transparent microwave metasurfaces with broadband absorption. However, the design of graphene-based microwave metasurfaces relies on cumbersome parameter sweeping as well as the expertise of researchers. In this paper, we propose a machine-learning network which enables the forward prediction of reflection spectra and inverse design of versatile microwave absorbers. Techniques such as the normalization of input and transposed convolution layers are introduced in the machine-learning network to make the model lightweight and efficient. Particularly, the tunable conductivity of graphene enables a new degree in the intelligent design of metasurfaces. The inverse design system based on the optimization method is proposed for the versatile design of microwave absorbers. Representative cases are demonstrated, showing very promising performances on satisfying various absorption requirements. The proposed machine-learning network has significant potential for the intelligent design of graphene-based metasurfaces for various microwave applications.

## 1. Introduction

Metasurfaces, composed of periodic or quasi-periodic two-dimensional (2D) arrays of subwavelength units, have emerged as one of the most thriving types of artificial electromagnetic surfaces, owing to their fascinating and tailorable electromagnetic properties [1,2]. In contrast to traditional bulk metamaterials [3,4,5,6], metasurfaces exhibit extreme thicknesses which enable engineering electromagnetic waves in phase, amplitude, and polarization through a compact and easiley fabricated system, providing great freedom in manipulating light-matter interactions at the sub-wavelength scale [7,8]. Such promising approaches prove their feasibility in numerous applications, from basic devices of holograms [9], electromagnetic absorbers [10], and polarizers [11], to more complex systems of information encryption [12,13], signal processing [14], and intelligent recognization [15].

Microwave absorption is one of the most important applications of metasurfaces, which are extremely useful in various engineering aspects [16,17]. Metasurface absorbers [18,19,20] can provide devisable bandwidth, ultra-thin thickness, and angular robustness, as compared to conventional microwave-absorbing materials or devices. Combining nanomaterials and metasurfaces provides a brand-new solution for excellent microwave absorption performance with optical transparency [21,22]. Recent advances in the study of 2D materials, particularly graphene, provide a novel viewpoint for the active control of electromagnetic waves throughout a wide spectrum [23,24]. Graphene possesses remarkable physical properties including monoatomic thickness, optical transparency, and unique electrical tunability attributable to its gapless and symmetrical band structure [25,26]. Notably, the electrostatic control of carrier concentration in graphene allows the dynamic manipulation of electromagnetic waves by adjusting graphene’s Fermi energy [27,28]. For example, garphene has been experimentally implemented for microwave absorbers [21,29]. Most recently, Zhang et al. [30] proposed an optically transparent and flexible microwave metasurface absorber based on a patterned graphene sandwich structure, which can achieve dynamic microwave absorption according to different bias voltages.

However, the traditional design process of graphene-based metasurfaces, including the design of the graphene pattern, the thickness of the dielectric layer, and the sheet resistance of graphene, depends on the expertise of researchers and time-consuming numerical simulations. The design of such metasurfaces demands a working knowledge base in order to moderate iterative simulations that scan multi-dimensional parameter spaces. In recent years, with the development of machine-learning methods and a burst in computation power from GPU acceleration, the concept of artificial intelligence (AI) has been introduced and applied in various research areas, such as image classification [31], natural language processing [32], and wireless communication [33]. It is also a popular interdisciplinary subject to introduce machine-learning technology as a tool in assisting the efficient and rapid design of metasurfaces [34,35,36]. Various neural networks have been used for designing metasurfaces, including multilayer perceptron (MLP) [37], deep neural networks [38], convolutional neural networks (CNN) [39], auto-encoders [40], and generative adversarial networks [41]. Machine learning is also helpful in designing metasurface absorbers. The forward prediction of the reflective spectra and inverse design of the microwave absorption metasurfaces could be achieved by building different machine-learning models. For example, variational autoencoder and covariance matrix-adaptation evolution strategies are utilized to find the optimal absorber in the X band [40]. Recent research about the intelligent design of metasurfaces that utilize machine-learning methods are focusing on the geometrical design. The design degrees are typically several geometrical parameters or the coding pattern of the resonant structures. The electromagnetic properties of materials are not considered in these works since the intrinsic material characteristics are not changeable in traditional design. However, the tunability of graphene enables a new degree of intelligent metasurface design. The sheet resistance of patterned graphene can be adjusted by bias voltage, which is used as a new design degree in this paper. In another aspect, the machine-learning models used in those studies are large and typically contain huge numbers of trainable parameters, some of which are actually redundant. However, the model could be designed specifically to contain a suitable number of trainable parameters. In that case, we call it a lightweight model, which is easy to train and also performs well efficiently.

In this paper, a lightweight machine-learning model is proposed and trained to predict the absorption spectrum of a graphene-based metasurface in milliseconds by putting in geometrical parameters of the patterned graphene layer and the tunable sheet resistance of graphene. Transposed convolution layers are adopted in the network to increase the performance of forward prediction and inverse design systems with the number of training parameters reducing at the same time. An inverse design system is constructed to give the optimized absorption result within the sampling space after specifying design requirements. This system combines the knowledge of the trained machine-learning model and optimization method to achieve quick and efficient design, which gives the optimized spectrum results, the optimized geometrical parameters, and the sheet resistance of graphene at the same time, within seconds.

## 2. Method

### 2.1. Graphene-Based Metasurface Absorber Model

The microwave metasurface absorber studied in this article by intelligent design consists of patterned graphene sandwich structures [30]. The top layer of the absorber is a graphene sandwich structure, which is based on a polyethylene glycol terephthalate (PET) substrate with a dielectric constant of 3. A thin ITO ground set is the bottom layer. It is worth noting that all those materials are optically transparent, so that the metasurface made of such materials would be optically transparent. This structure utilizes graphene’s dynamic conductivity by applying different bias voltages and can be used for tunable broadband absorption. The graphene layer is modeled as an infinitesimally thin resistive surface characterized by a sheet resistance $R_{g}$ given by the well-established Kubo formula [42]. The bias voltage directly changes the sheet resistance of the patterned graphene layer, resulting in the dynamic change in the absorption performance at different frequency ranges. The sheet resistance of the graphene layer can be simplified as [43]:

(1) $$R_{g}=\frac{1}{\sigma_{g}}\approx \frac{\pi \hbar^{2}\left(\omega +i2\Gamma \right)}{ie^{2}k_{B}T}{\left[\frac{E_{F}}{k_{B}T}\;+\;2\ln\left(1\;+\;e^{\frac{E_{F}}{k_{B}T}}\right)\right]}^{-1}$$

where *e* represents the electron charge constants, and *ℏ* and $k_{B}$ are the Planck’s and Boltzmann’s constants, respectively. ω represents the operation angular frequency. *T* is the room temperature and $E_{F}$ is the Fermi energy of graphene proportional to the external bias voltage. ℏ=1/2τ is the phenomenological scattering rate(τ is the electron-phonon relaxation time). We consider T=300 K and τ=0.2 ps. The electromagnetic performance of such a metasurface absorber critically relies on the patterned graphene layer [26,44]. By modifying the geometry of the patterned graphene layer and the sheet resistance of graphene, versatile absorption performances can be obtained. However, the conventional design of metasurface structures relies on massive numerical simulations for computing the electromagnetic response of different parameter combinations, which is time-consuming and redundant. In this paper, we utilize neural networks and suitable machine-learning techniques to propose an efficient, user-friendly, and high-performance design system for graphene-based metasurface absorbers.

### 2.2. Machine-Learning Model

**Figure 1:**
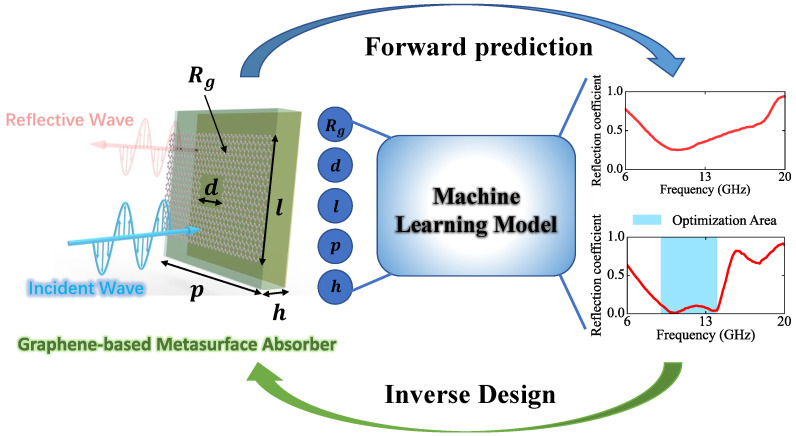
Schematic diagram of forward prediction and inverse design of graphene-based microwave absorbers using machine-learning model.

We propose the machine-learning prototype utilizing an MLP network and a transposed convolution technique to realize the fast prediction of reflection coefficients in the range of 6–20 GHz, according to the combination of several geometrical parameters. In our work, a combination of 5 parameters is used as the input information and the reflection coefficients can be inferred as the output of the machine-learning model. There are 4 geometrical parameters, p,d,l,h. *p* represents the period of meta unit, *d* is the length of the middle square hole, *l* is the length of graphene in the y-axis, and *h* is the thickness of the PET substrate, as shown in Figure 1. The tunable sheet resistance $R_{g}$ of the graphene layer is another parameter considered in the machine-learning model.

Reflection coefficients are points evenly taken from the results of numerical simulations via CST Microwave Studio, corresponding to the combinations of 5 parameters. A total of 281 points in the range of 6–20 GHz are used for approximation. Therefore, every sample consists of two vectors from the linear space of $R^{5}$ and $R^{281}$, respectively.

Samples in the dataset are uniformly distributed from a reasonable range of the linear space $R^{5}$, which restricts those parameters not to violate topology and are in accordance with physics. Moreover, all parameters are normalized to [0,1] before being put into the model, as Equation (2) illustrates:

(2) $$\bar{s}=\frac{s}{s_{max}-s_{min}}$$

where *s* represents the value of any of the 5 parameters, $\bar{s}$ represents the normalized value, $s_{max}$ is the maximum value of this parameter while $s_{min}$ is the minimum value. Normalization reduces the impact of numerical differences between different parameters and makes the training process more stable and effective. Thus, the excellent performance of the forward prediction network is guaranteed when taking those 5 normalized parameters as input.

The convolution techniques that are adopted in CNN are used in lots of fields such as digital image and voice processing [45,46]. They can combine the information in local fields in learning and work as feature-extraction tools. The convolution operation decreases the spatial dimensions and produces an abstract representation of the input image as we go deeper down the network [47]. Recent research into inverse design of metasurfaces uses 1D convolution to extract spectrum features and build inverse model to predict design parameters directly [48]. Here, we add transposed convolution techniques to our forward prediction network to serve the inverse design system better. Transposed convolution is also known as deconvolution, which is not appropriate as deconvolution implies removing the effect of convolution, which we are not aiming to achieve. It is used as a efficient upsampling tool in the modern image semantic segmentation [49,50] and super-resolution algorithms [51]. Deconvolution can also be used to observe the feature-learning performance of the intermediate convolution layer, and is mostly used in image processing and pattern recognition. In our work, since the network model is finally used for inverse design, transposed convolution techniques are used to upsampling the reshaped features from hidden layers and the reconstruct reflective spectrum, which improve the effect of inverse design, to a certain extent.

**Figure 2:**
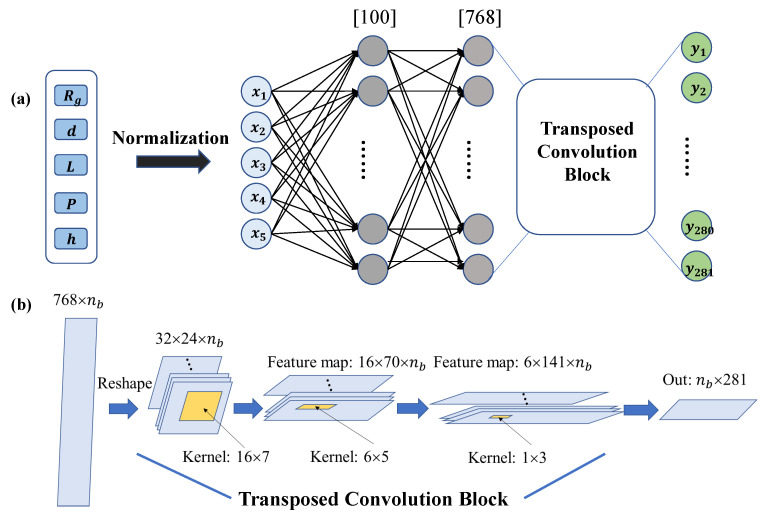
Illustration of forward prediction machine-learning network. (**a**) The five design parameters are normalized as a 5-dimension vector input for the machine-learning network. The output points correspond to 281 sample points of reflective spectrum in 6–20 GHz. The network consists of two fully connected layers and a transposed convolution block. (**b**) The working mechanism of transposed convolution block considering batch size in training process with three transposed convolution layers. Here, $n_{b}$ is the batch size. The kernel size for each layer and feature-map evolution are demonstrated. This block eventually turns information from the second fully connected layer into 281 dimensions’ output which can reproduce the reflective spectrum.

That strategy also helps improve the performance of prediction in some boundaries of input sampling space and reduce the training parameters of this machine-learning model. That is, we make the model more lightweight and easier to train without performance degradation by introducing transposed convolution layers. The architecture of our deep-learning network is shown in Figure 2. There are two fully connected layers with 100 neurons and 700 neurons in the linear block and three 1D transposed convolution layers in our transposed convolution block. Neurons in the linear block are activated by the Leaky ReLU (rectified linear unit) activation function while those in transposed convolution block are activated by the ReLU activation function.

The total number of trainable parameters in our model is 82,250, which is significantly less than models from recent research on forward predicting light spectrum by AI. The training process of this network is the optimization of a loss function. Minimum square error (MSE) loss is a simple and suitable loss function for our network, which is defined as:

(3) $$L_{\mathrm{MSE}}=\frac{1}{2n}\sum_{i}^{n}{\left({\hat{y}}^{i}-y^{i}\right)}^{2},$$

with ${\hat{y}}^{i}$ denoting the output of MLP network when the input is $x^{i}$, and *n* denoting the amount of training samples.

We call this machine-learning model a forward prediction network (FPN), which can perform accurate simulation replacing numerical electromagnetic simulations. The training progress is discussed in Section 3.2. The FPN learns knowledge of electromagnetic theory from data and reproduces the calculation correctly gradually.

### 2.3. Inverse Design System

For inverse design, the FPN model can be seen as a black-box function. That is, the trained FPN model is used as a function F(x), defined as:

(4) $$\begin{array}{cc} \; & x\overset{F}{\to }y,\\ \; & x\in R^{5},y\in R^{281}. \end{array}$$

**Figure 3:**
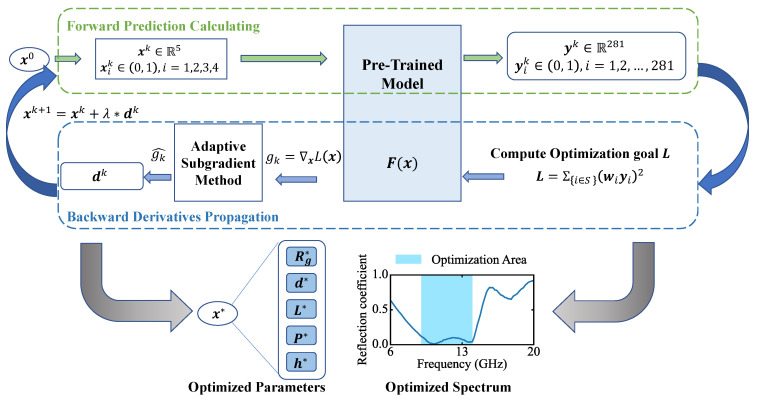
Illustration of inverse design system optimization process. Before training begins, an initial seed $x_{0}$ is generated randomly. In iterative step *k*, loss *L* and the its gradients to x: $g_{k}=\nabla_{x}L\left(x\right)$ are firstly computed through the pre-trained model. ${\hat{g}}_{k}$ is derived by the adaptive subgradient method [52] based on $g_{k}$ in all iterations before k-th to determine the desecending direction $d^{k}$, which is the same dimension as x. λ is the descending step of each iterative, which is a scalar in (0, 1). x is updated with the iterative paradigm. After a fixed number of iterative steps, the optimized result can be then solved. Combinations of optimized parameters of any design requirements can be obtained within seconds.

Obviously, F is continuous when x∈X, and the input space is 5 dimensional and the output space is 281 dimensional. Partial derivatives $\partial y_{i}/\partial x_{j}$ exist and can be calculated for every i=1,2,…,281 and j=1,2,…,5. Thus, F is the first-order differentiate. F(x) is an abstract function, not like a normal sine or polynomial function that can be mathematically expressed easily. We only need to know the input and output, while the relationship between input and output is learned from the machine-learning training process, which is also called “knowledge” of AI. We can utilize this “knowledge” for our further research and do not need to understand its fundamentals. The Jacobi matrix of $F(\bar{x})$ for $\forall \bar{x}\in X$ can be easily calculated numerically by back propagation using machine-learning toolbox. Since we need to design a specific absorber, that means we have some pre-defined requirements on the absorption spectrum. This can also be represented by a reflective spectrum, since the transmissive wave is neglectable in our design. In the absorber design, we typically want the band of absorption to be as wide as possible, or the intensity of absorption to be as strong as possible. Based on such requirements of absorber design, we can set optimization goals on the reflective spectrum according to different requirements. The optimization goal *L* can be set as:

(5) $$L=\sum_{x_{i}\in S}{\left(w_{i}F{\left(x\right)}_{i}\right)}^{2},$$

where *S* denotes the points set of the designed absorption frequency range, and $F{\left(x\right)}_{i}$ represents the *i*-th element of the output vector y=F(x). Here, $w_{i}$ is the optimization weight of $F{\left(x\right)}_{i}$, which can fine tune the optimization results. The optimization problem can be presented as:

(6) $$\begin{array}{cc} \; & \min_{x}\sum_{x_{i}\in S}{\left(w_{i}F{\left(x\right)}_{i}\right)}^{2}\\ \; & s.t.\;x\in X \end{array}$$

where *X* is the input space of our FPN. Therefore, fulfilling the absorber design requirements turns into solving an optimization of the first-order differentiable continuous functions with constraints. Selecting an optimization algorithm in the conventional convex optimization field such as steepest descent, conjugate gradient or Lagrange multiplier method, and penalty function method can make our inverse design system work. Since the Jacobi matrix of F(x) can be obtained directly from the machine-learning toolbox, the optimization process can also be performed in a machine-learning prototype effectively. The absorption in the frequency range *S* can reach the optimized state by minimizing *L*. The working mechanism is shown in Figure 3, where $\nabla_{x}L\left(x\right)$ is computed by Chain rule in calculus:

$$\begin{array}{c} \nabla_{x}L\left(x\right)=\left(\begin{array}{c} \frac{\partial L}{\partial x_{1}}\\ \frac{\partial L}{\partial x_{2}}\\ \vdots \\ \frac{\partial L}{\partial x_{5}} \end{array}\right)=\left(\begin{array}{c} \sum_{i=1}^{281}\frac{\partial L}{\partial y_{i}}\cdot \frac{\partial y_{i}}{\partial x_{1}}\\ \sum_{i=1}^{281}\frac{\partial L}{\partial y_{i}}\cdot \frac{\partial y_{i}}{\partial x_{2}}\\ \vdots \\ \sum_{i=1}^{281}\frac{\partial L}{\partial y_{i}}\cdot \frac{\partial y_{i}}{\partial x_{5}} \end{array}\right). \end{array}$$

To implement the inverse design system, we define a new machine-learning model with nearly the same structure as the forward prediction model. The new model does not need input as FPN. The first layer of the new model is working as model input, but is trainable instead. After the first layer, the network architecture is the same as our pre-trained FPN model. Then, we fix the weight and bias in the new model except for the first layer and set those quantities to exactly the same as pre-trained FPN model, which means these parts work as the black-box function F(x). In the training process, the parameters of the first layer are optimized by our optimization method while other parameters of the new machine-learning model remain unchanged.

In the inverse design system building, we realize the differentiable property of the machine-learning model and utilize the model as a black-box function. CST generates data in a complex numerical calculation manner while the machine-learning model can mine the implicit knowledge contained in the generated data. Therefore, for this graphene-based metasurface absorber, the principle of numerical calculation can be concisely and accurately represented by machine learning, which is crucial to establishing a fast and effective inverse design system. The insights gained from machine learning have great potential to expand to other nanomaterials applications.

## 3. Experiments and Results

### 3.1. Dataset Collection

**Figure 4:**
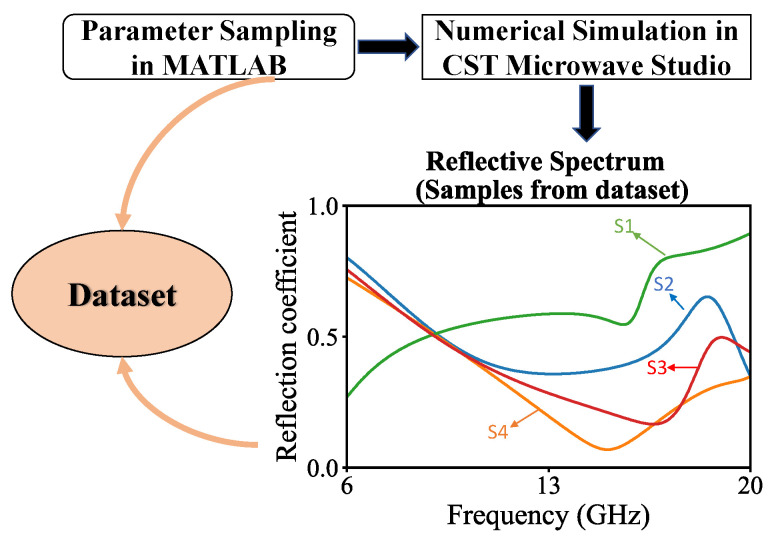
Illustration of dataset generation. S1, S2, S3, S4 are randomly selected sample spectra from dataset.

To train the forward prediction neural network, we first need a dataset of our graphene-based metasurface with adequate data to sample space. Simulations for 7000 combinations of parameters are conducted by Matlab-CST co-simulation. The metasurface is first modeled and the simulation condition is set in commercial software CST Microwave Studio. In the simulations, periodic boundary conditions were set along the *x* and *y* directions and the Flouquet port excitation was applied along the *z* direction. In the process of the generation of data, parameter combinations are firstly uniformly sampled in Matlab. The built-in Visual Basic interface of CST is utilized in Matlab to change the dynamic parameters that we need. In each iteration, CST Microwave Studio fetches one parameter combination from Matlab, runs the corresponding numerical simulation, and passes calculated spectrum data back to Matlab. The values of parameters are uniformly sampled from constrained space, which keeps the topology and physical rules correct. In this way, 7000 pairs of data containing parameter combinations and spectrum are finally generated and organized into the dataset. The sampling process and the range of parameters are shown in Figure 4 and Table 1.

### 3.2. Performance of Forward Prediction

**Figure 5:**
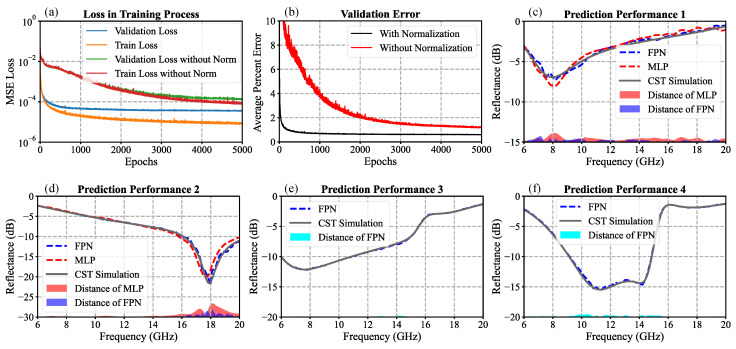
(**a**) Train and validation loss in the training process with and without normalization. (**b**) Average percentage error of each 281 spectrum-point visualizations in the training process. (**c**,**d**) Prediction performance with and without transposed convolution layers in two extreme situations when the parameters are at the boundaries of sampling space. (**e**,**f**) Two examples of prediction performance of our FPN model.

The hyperparameters for training models are shown in Table 2, which is not heavily optimized but can achieve our objectives. The training process is achieved in the online platform Kaggle with GPU acceleration by Nvidia Tesla P100. This GPU has the NVIDIA Pascal GPU architecture, which is optimized to support novel deep-learning applications. Figure 5a shows the loss of training and validation data during the training process, for the cases with and without normalization of the input parameters. It can be seen that the train loss with normalization converges to below $10^{-5}$, one order lower than the one without normalization. The validation loss with normalization also converges to $3\times 10^{-5}$, much lower than the one without normalization, $1.37\times 10^{-4}$. Moreover, both the validation and the train loss with normalization drop very quickly in the first 1000 epochs, much faster than those without normalization. The comparison of model training with and without normalization shows that the normalization of the input parameters speeds up the convergence of loss and makes the test error significantly lower. The average percent error for each spectrum is defined as the difference between the prediction of the FPN model and the ground truth from CST simulation, divided by the latter. Figure 5b shows the average percent error with and without normalization. It can be seen that the error for each spectrum point is less than 1% after 1000 epochs of training with normalization, which shows the extraordinary prediction accuracy of our FPN models.

To better show the performace of our network architecture, we build an MLP model for comparison. The MLP model has two hidden layers, the same as our FPN, with a fully connected last hidden layer with a 281-dimension vector output. The nodes of the four layers (including input and output) are 5, 100, 700, 281. Therefore, the total trainable parameters are 268,281, about 4 times our FPN model. We also define a measurable criterion to evaluate the performance of different models more visually. The distance D refers to the error of one prediction of the model defined as:

(7) $$D=|10\log\left(\hat{y}\right)-10\log\left(y\right)|.$$

Figure 5b shows the prediction of the reflective spectrum by models with and without transposed convolution layers. It is clear that, in some extreme situations, the distance of our FPN remains quite low while the MLP performs poorly in some frequencies.

The model is trained well quite quickly, with less than 25 min required to achieve very promising accuracy for the forward prediction. The forward-prediction performance of evaluating examples not used for model training is shown in Figure 5e,f. The our FPN can run thousands of simulations in milliseconds and its result perfectly matches the ones of CST simulation. Owing to its concise and lightweight architecture and extremely promising prediction precision, our model is able to be trained quickly and efficiently. Once we collect some minor datasets in other frequency regimes with specifying geometries, the model is also scalable and valid across terahertz or infrared regimes by transfer learning, which is worth further investigation.

### 3.3. Results of Inverse Design System

The absorptivity A(ω) of the proposed metasurface can be calculated by:

(8) $$A\left(\omega \right)=1-{\left|r\left(\omega \right)\right|}^{2}-{\left|t\left(\omega \right)\right|}^{2}\approx 1-{\left|r\left(\omega \right)\right|}^{2}.$$

Here, r(ω) and t(ω) are the reflection and transmission coefficients, with t(ω) being negligibly low.

**Figure 6:**
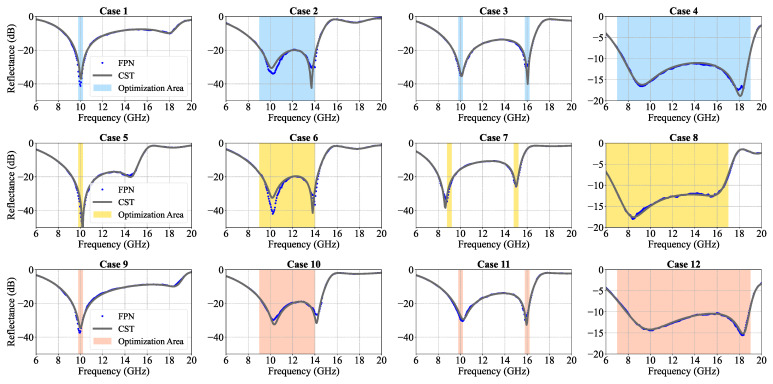
Versatile absorber designs based on different requirements. (The reflectance are shown in logarithmic coordinate) Cases 1–4: inverse design in all five degree of freedom. Cases 5–8: inverse design with thickness of substrate *h* fixed at 3.5 mm. Cases 9–12: inverse design with sheet resistance of graphene $R_{g}$ fixed at 250 Ω. The colored area is the optimization area. FPN indicates the optimized reflection spectra given by inverse design system while CST represents the reflection spectra from CST simulations with the optimized parameter combinations.

As a first example, we set the targeted frequency at around 10 GHz to find the highest absorptivity as possible, as shown in Case 1 of Figure 6. It is seen that a minimum reflectance reaching −50 dB could be optimized at 10 GHz, reaching a peak absorption over 99.99%. As a second example, the absorption frequency range is targeted as 9–14 GHz, as shown in Case 2 of Figure 6. After optimization, a wide-band absorption spectrum can be obtained and more than 99% absorptivity achieved in the entire band of 9–14 GHz. We can also set two targeted frequencies separately as the optimization regions for seeking a metasurface absorber with dual-band absorption. As shown in Case 3 of Figure 6, there is nearly 99.9% absorption in the dual peaks at 10 GHz and 16 GHz. In Case 4 of Figure 6, we show an ultra-wide band absorption optimization, where over 90% absorption have been achieved within the frequency band 7.55–18.8 GHz, covering both X-band and Ku-band.

Figure 6 shows the reflective spectrum of FPN prediction and the CST simulation result based on optimized parameter combinations. The corresponding parameter combinations are shown in Table 3, verifying the effectiveness and performance of our machine-learning model based the inverse design of versatile metasurface absorbers. Those cases are chosen from arbitrary given requirements, which reveal the universal applicability of our inverse design system.

In most situations, the dielectric substrates are typically of standard thicknesses. Substrates with non-standard thicknesses are hard to fabricate or of high cost. If, for example, we fix the thickness *h* to be 3.5 mm, the design system can still give promising results. In Figure 6, Cases 5–8 show the optimization with the remaining four design parameters to achieve the required absorption performance. It is seen that most requirements can still be satisfied well. Since the thickness of substrate h=3.5 mm is relatively thick and affects the resonance frequency evidently, the absorption frequency bands in Cases 7 and 8 show a slight red shift as compared to those in Cases 3 and 4.

On the other hand, since the tunable patterned graphene is more difficult to fabricate than unchangeable graphene, it is also meaningful to examine the performance of our inverse design system with the fixed-sheet resistance of graphene. Here, assuming the graphene layer has a fixed-sheet resistance of 250 Ω, different absorbers can still be designed well. Figure 6 Cases 9–12 show the inverse design results by optimizing the remaining four geometrical parameters while fixing the sheet resistance of graphene. We can see that the absorption performances in Cases 9–12 are obtained very similarly to those of Cases 1–4, with little degradation in absorptivities. All these results indicate that our inverse design system has good design flexibility. The satisfactory performance of our model indicates its potential in other nanomaterials applications. When it comes to a new material system, the reasonable parameter-sampling space should be given, firstly, according to professionals of engineers. Then, similar training procedures and inverse-design-system building could be applied, as such a model is scalable to other material systems.

## 4. Conclusions

In this work, we proposed a novel machine-learning-model-based inverse-design system for designing graphene-based metasurface absorbers with versatile absorption performance. Transposed convolution layers were introduced in our forward-prediction architecture for reducing the model size, which improves performance. With the key parameters of the metasurface normalized as input, the forward-prediction model can quickly predict the reflective spectra of the absorbers with high accuracy, as compared to numerical simulations. Based on the well-trained machine-learning model, we built an inverse-design system to optimize versatile-absorption performance. Given the optimization goal for specified absorption frequencies, the system can find the optimized results in a sampling space in seconds. The insights gathered from this paper could help with the intelligent design for other type of graphene-based metasurfaces or devices.

## Figures and Tables

**Table 1:** Design parameters sampling space.

Design Parameters	Start	End
$R_{g}$ (Ω)	132	300
*d* (mm)	1	6
*l* (mm)	5	11
*p* (mm)	8	14
*h* (mm)	2	4

**Table 2:** Hyperparameters in model training.

Hyperparameters	Values
Learning rate	3 × 10${}^{-4}$
Optimization method	Adam
Learning-rate decay	5 × 10${}^{-6}$
Loss function	MSELoss

**Table 3:** Parameters combinations for cases in Figure 6.

Parameters	$R_{g}$ (Ω)	*d* (mm)	*l* (mm)	*p* (mm)	*h* (mm)
Case 1	136.42	1	6.36	11.63	2.6
Case 2	149.48	5.29	7.23	14	3.57
Case 3	153.8	3.43	6.49	12.26	3.14
Case 4	132	3.73	7	10.62	2.91
Case 5	171.48	4.42	7.58	13.17	3.5
Case 6	154.39	4.85	7.04	14	3.5
Case 7	179.47	2.38	7.35	13.18	3.5
Case 8	132	4.75	7.58	11.38	3.5
Case 9	250	1	6.28	10.43	3.16
Case 10	250	2.3	7.3	14	3.41
Case 11	250	1	6.86	12.5	3.16
Case 12	250	1.11	7.72	10.84	2.83
